# Preliminary report on embolization with quick-soluble gelatin sponge particles for angiographically negative acute gastrointestinal bleeding

**DOI:** 10.1038/s41598-024-56992-5

**Published:** 2024-03-18

**Authors:** Meshari Alali, Chuanwu Cao, Ji Hoon Shin, Gayoung Jeon, Chu Hui Zeng, Jung-Hoon Park, Shakir Aljerdah, Sultan Aljohani

**Affiliations:** 1 Department of Radiology, Majmaah University, Almajmaah, Saudi Arabia; 2 Department of Radiology, The Tenth People’s Hospital, Shanghai, China; 3 Department of Radiology, University of Ulsan College of Medicine, Asan Medical Center, Olympic-ro 43-gil, Songpa-gu, Seoul, 05505 Korea; 4 Department of Radiology, Najran University, Najran, Saudi Arabia; 5 Department of Radiology, King Salman Bin Abdulaziz Medical City, Medina, Saudi Arabia

**Keywords:** Gastroenterology, Medical research

## Abstract

Prophylactic embolization is usually performed using gelatin sponge particles, which are absorbed within several weeks, for managing angiographically negative gastrointestinal bleeding. This study aimed to evaluate the safety and effectiveness of transcatheter arterial embolization (TAE) with quick-soluble gelatin sponge particles (QS-GSP) that dissolve in less than 4 h for treating angiographically negative gastrointestinal bleeding. We included ten patients (M:F = 7:3; mean age, 64.3 years) who underwent prophylactic TAE with QS-GSP for angiographically negative acute gastrointestinal bleeding between 2021 and 2023. The technical success rate of TAE, clinical outcomes focusing on rebleeding, and procedure-related complications were evaluated. The embolized arteries were the gastroduodenal (n = 3), jejunal (n = 4), and ileal (n = 3) arteries. QS-GSP (150–350 µm or 350–560 µm) were used alone (n = 8) or in combination with a coil (n = 1). A 100% technical success rate was accomplished. In 1 patient (10%), rebleeding occurred 2 days after prophylactic TAE of the gastroduodenal artery, and this was managed by repeat TAE. There were no procedure-related complications. The use of QS-GSP for prophylactic TAE appears to be safe and effective for controlling bleeding among patients with angiographically negative gastrointestinal bleeding. There were no cases of related ischemic complications of the embolized bowels likely attributable to recanalization of the affected arteries following biodegradation of QS-GSP.

## Introduction

While endoscopy serves as the primary approach for diagnosing and managing gastrointestinal (GI) bleeding, transcatheter arterial embolization (TAE) serves as a useful non-surgical treatment when the source of GI bleeding is either inaccessible by endoscopy or cannot be treated via endoscopy^1,2^. TAE for GI bleeding can be performed using a variety of embolic materials, and the clinical success rate is reported to be between 63 and 97%^3^. However, in acute GI bleeding, conventional angiography frequently fails to detect active bleeding. Previous research has indicated rates of negative angiographic findings ranging between 24 and 78%^4,5^.

Prophylactic embolization for angiographically negative GI bleeding (negative GI bleeding on conventional angiography) is known to be both controversial and effective, and the primary embolic materials have been gelatin sponge particles (GSPs) and coils^6–9^. Coils are permanent embolic materials and are considered unsuitable for prophylactic TAE because of the risk of coil migration; GSPs, on the other hand, take several weeks to be absorbed, which can lead to ischemia or infarction of the bowel^10,11^.

Therefore, it is thought that the risk of bowel ischemia or infarction can be reduced if GSPs are absorbed more quickly. Quick-soluble GSPs (QS-GSPs) which dissolve more rapidly than conventional GSPs, have been developed to provide embolization effective for several hours^12^. A recent study demonstrated that genicular artery embolization using these QS-GSPs was safe and resulted in significant pain reduction^12^. To date, there are no reports of QS-GSP use for treating GI bleeding; however, the safe application of QS-GSPs in prophylactic embolization for angiographically negative GI bleeding is being considered. This preliminary study aimed to evaluate the safety and effectiveness of prophylactic TAE using QS-GSPs for angiographically negative GI bleeding.

## Results

Clinical characteristics, TAE details, and clinical outcomes are summarized in the Table 1. The diagnosis of active bleeding was made using endoscopy (n = 3), CT (n = 2), or both (n = 2). In 3 patients, active bleeding was not found on endoscopy (n = 1) or CT (n = 2). Seven patients had previously undergone treatment for GI bleeding via the following strategies: endoscopic clipping with or without epinephrine injection (n = 4), TAE (n = 2), and small bowel resection with TAE (n = 1).

**Table 1:** Clinical characteristics, procedure details, and clinical outcomes of 9 patients who underwent prophylactic TAE with QS-GSP.

No./Age/Sex	Cause of bleeding	Underlying diseases	Interval	CM	Diagnostic modality	Previous treatment	Embolized arteries	Embolic materials	Hb (g/dL)		Outcome (FU duration)	Remark
									Pre	Post		
1/68/M	Jejunal ulcer	CKD, DM, HTN, Pontine infarct	6 days	Melena	EGD (SD)- jejunal ulcer with bleeding	Endoscopic clipping and epinephrine (SD)	SMA jejunal br	QS-GSP (150–350 µm)	7.9	9.1	No further bleeding (27 months)	Vasa recta level embo. near clips
2/63/M	Duodenal ulcer	Pancreatic ca., HTN	7 days	Hemato-chezia	CT (1DP)- duodenal ulcer	No	GDA	QS-GSP (350–560 µm)	7.5	8.9	Rebleeding 2 days later	PSA of supraduodenal a. – embolized with NBCA/coil 5 days later
3/63/F	Jejunal ulcer	Behcet’s disease	1 day	Hemato-chezia	CT (SD)- active jejunal bleeding	Small bowel resection 9 MA, Embo. for jejunal br. 2 MA	SMA Jejunal br	QS-GSP (350–560 µm)	8.3	9.5	No further bleeding (22 months)	Segmental resection of necrotic pelvic ileum 2 weeks later
4/57/F	Duodenal ulcer	CKD (KT), DM, ACS, coagulopathy	1 day	Hema-temesis*	EGD (SD)- duodenal 2nd portion ulcer with hematoma, CT (SD) – active bleeding	Endoscopic clipping (SD)	GDA br	1 coil & QS-GSP (150–350 µm)	6.1	10.1	No further bleeding until death	Right radial a. due to ECMO state, died 4 days later (cardiogenic shock)
5/60/M	Ileal metastasis bleeding	HCC with metastasis	2 weeks	Melena*	CT (1DP)- peritoneal/bowel seeding, no active bleeding	Embo. for duodenal metastasis 1 YA	Ileal br	QS-GSP (150–350 µm)	6.5	7.8	No further bleeding until death	Died 5 weeks later (cancer progression)
6/72/M	Lymphoma involving ileum	DLBCL, coagulopathy	1 day	Hemato-chezia	CT (1DP)- no bleeding, EGD (SD) – ileal ulcer with oozing bleeding	Endoscopic clipping and epinephrine (SD)	Ileal br	QS-GSP (350–560 µm)	8.4	9.6	No further bleeding (18 months)	PSA-like contrast pooling could not be selected, so, possible feeders embolized with QS-GSP
7/47/F	Jejunal metastasis bleeding	Uterine leiomyo-sarcoma	3 days	Hemo-peritoneum	CT (3DP)- peritoneal seeding with internal bleeding	No	Jejunal br	QS-GSP (350–560 µm)	6.8	8.7	No further bleeding (surgery followed)	Peritoneal seeding removal 2 days later, died 4 months later (cancer progression)
8/75/M	Duodenal ulcer	Duodenal ulcer	4 days	Hema-temesis, melena	CT (SD)- no bleeding focus, EGD (3DP)- active duodenal bulb bleeding	Endoscopic clipping (3DP)	GDA br	QS-GSP (150–350 µm)	6.6	8.5	No further bleeding (10 months)	
9/67/M	Terminal ileal ulcer	Behcet’s disease, CKD, DM	10 days	Hemato-chezia	CFS (3DP)- terminal ileal ulcer	Endoscopic clipping (3DP)	Ileal br	QS-GSP (350–560 µm)	7.2	8.9	No further bleeding (3 months)	
10/71/M	Jejunal ulcer	HTN	2 days	Hemato-chezia	CT (1 DP)- active jejunal bleeding, EGD (SD)- jejunal ulcer	No	Jejunal br	QS-GSP (350–560 µm)	7.2	12.3	No further bleeding (1 month)	s/p PPPD for common bile duct cancer (3 years before)

*Hemodynamic instability was present. CKD = chronic kidney disease; DM = diabetes mellitus; HTN = hypertension; CM = clinical manifestation; ca. = cancer; ACS = acute coronary syndrome; HCC = hepatocellular carcinoma; DLBCL = diffuse large B-cell lymphoma; EGD = esophagogastroduodenoscopy; SD = same day; DP = day(s) prior; CFS = colonofiberscopy; MA = months ago; embo. = embolization; SMA = superior mesenteric artery; br. = branch; GDA = gastroduodenal artery; QS-GSP = quick-soluble gelatin sponge particles; Hb = hemoglobin; FU = follow-up; PSA = pseudoaneurysm; NBCA = n-butyl cyanoacrylate; ECMO = extracorporeal membrane oxygenation.

The main clinical manifestations were hematochezia (n = 5), melena (n = 3), and hematemesis (n = 2). Bleeding sites were in the duodenum (n = 3), jejunum (n = 4), and ileum (n = 3). The causes of bleeding were ulceration (n = 7), metastasis to the jejunum or ileum (n = 2), and lymphoma involving the ileum (n = 1). The median interval between the onset of GI bleeding and TAE was 2.5 days (range, 1–14 days). Three patients (Patients 4, 6 and 10) had coagulopathy. Hemodynamic instability was observed in 2 patients (Patients 4 and 5).

**Figure 1:**
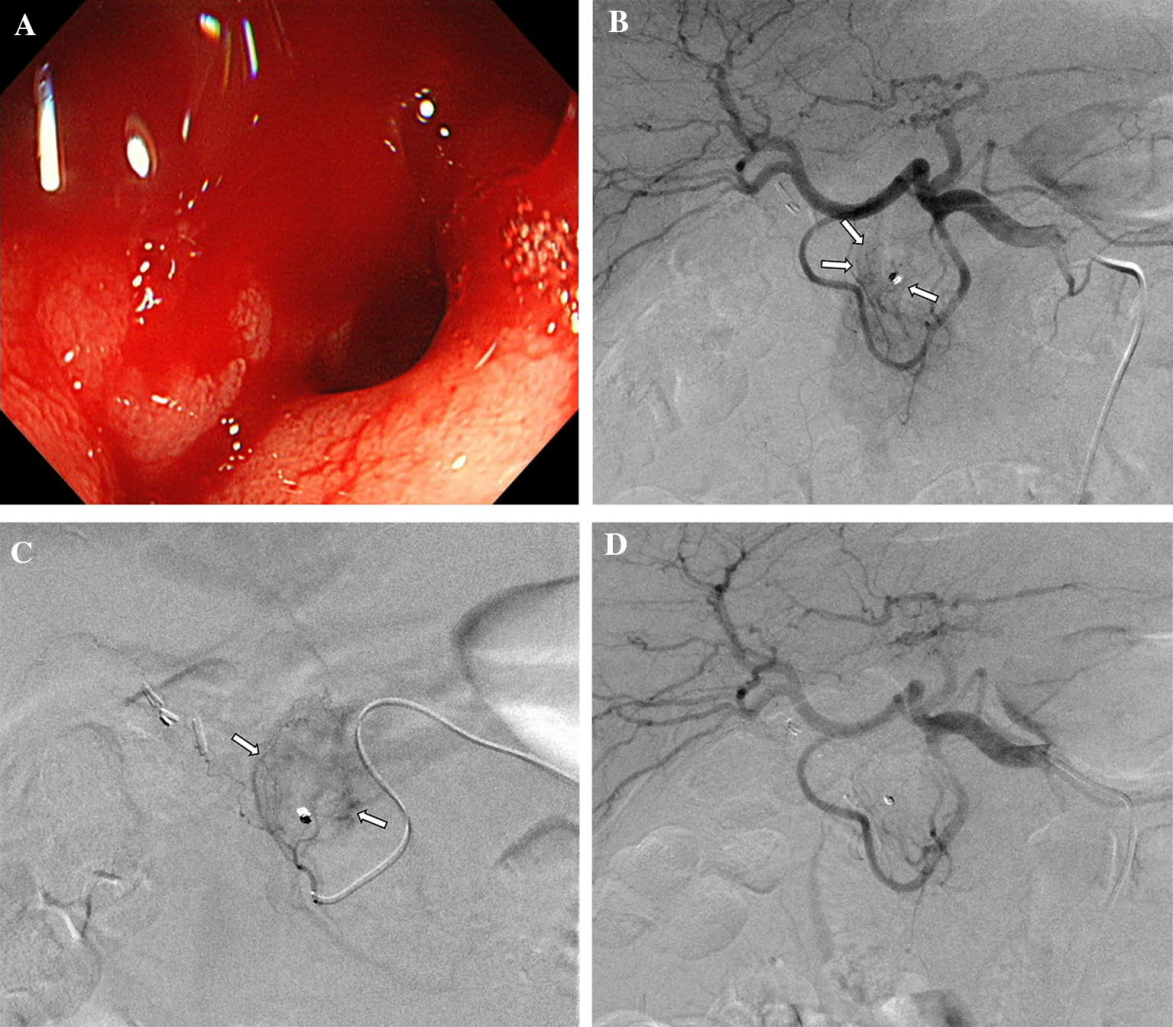
A 75-year-old man presented with hematochezia and melena 4 days prior (Patient 8). **(a)** Endoscopy shows active bleeding at the duodenal bulb. Blind hemoclipping was performed (not shown). **(b,c)** Angiography of the common hepatic artery **(b)** and duodenal branch of the gastroduodenal artery **(c)** 3 days after endoscopy shows no definite bleeding, but parenchymal blush (arrows) was evident around the hemoclips (arrows). The duodenal branch was embolized with quick-soluble gelatin sponge particles (150–350 µm) (not shown). **(d)** Completion common hepatic angiography shows no parenchymal blush around the hemoclips. He had no rebleeding during 10 months of follow-up.

**Figure 2:**
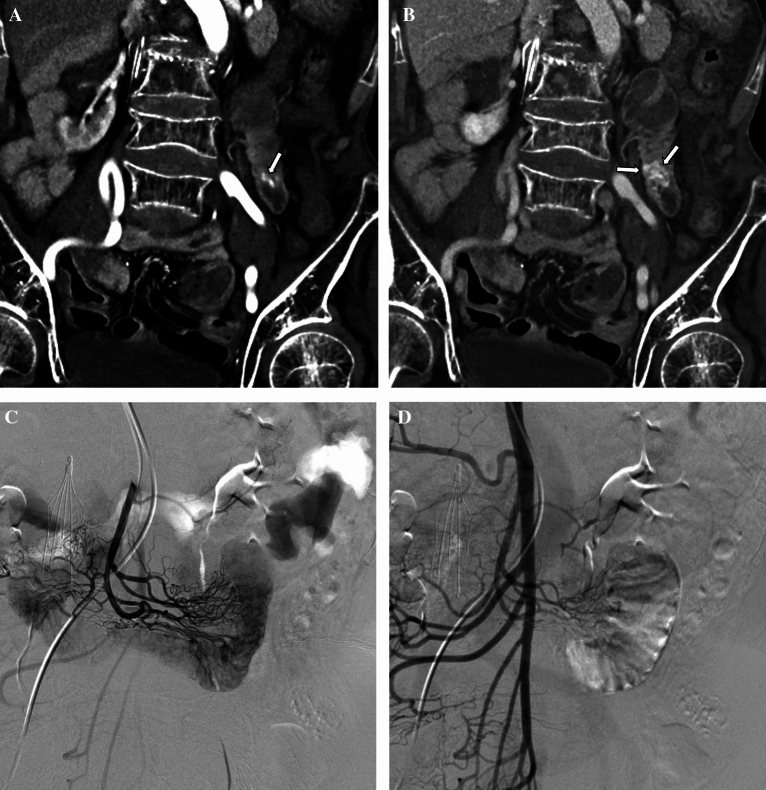
A 63-year-old woman with underlying Behçet’s disease presented with hematochezia 1 day prior (Patient 3). **(a,b)** Arterial **(a)** and venous **(b)** phases of computed tomography show active bleeding (arrows) in the jejunum. **(c)** Selective angiography through the superior mesenteric artery shows no definite bleeding focus at the jejunum matching the bleeding site indicated by computed tomography. These jejunal branches were embolized with quick-soluble gelatin sponge particles (350–560 µm) (not shown). **(d)** The embolized jejunal branches were not visible via completion superior mesenteric arteriography. She had no rebleeding during 22 months of follow-up.

The target artery for embolization was decided based on endoscopic and/or CT findings, with reference to angiography. In 1 patient (Patient 4), right radial access was used for TAE because both femoral arteries were cannulated for ECMO (extracorporeal membrane oxygenation). The embolized arteries were the gastroduodenal (n = 3, Fig. 1), jejunal (n = 4, Fig. 2), and ileal (n = 3) arteries. QS-GSPs (150–350 or 350–560 μm) were used alone (n = 9) or in combination with a coil (n = 1). The QS-GSP diameters were 150–350 μm (n = 4) or 350–560 μm (n = 6). Occlusion or stasis of flow in the target artery on angiography after TAE was achieved in all patients (Figs. 1,2), resulting in a 100% technical success rate.

Clinical success was achieved in 9 patients (90%). During a follow-up period of 1–28 months after TAE, 7 patients experienced no rebleeding. One patient (Patient 7) had no rebleeding until undergoing surgery for peritoneal seeding 2 days after TAE, and the other 2 patients had no rebleeding events prior to their deaths from cardiogenic shock (4 days after TAE) and cancer progression (5 weeks after TAE), respectively. The median hemoglobin level changed from 7.2 ± 0.78 to 9 ± 1.22 g/dL (*P* < 0.05).

Clinical failure in 1 patient (Patient 2, 10%) was due to rebleeding, which occurred 2 days after prophylactic TAE of the gastroduodenal artery. This rebleeding was managed by repeat TAE with NBCA (n-butyl cyanoacrylate) and microcoils, resulting in clinical recovery.

There were no procedure-related complications during follow-up. Ulceration and perforation of the pelvic ileal loop occurred 2 weeks after prophylactic TAE of the jejunum in 1 patient (Patient 3) who had previously undergone jejunal resection and TAE to treat jejunal bleeding caused by Behçet’s disease. The patient underwent segmental resection of this ulcerated pelvic ileal loop. The embolized jejunum was intact in the operating room.

## Discussion

TAE is effective when there is a GI bleeding focus. However, the absence of definitive guidelines for the use of prophylactic TAE in situations where a bleeding focus is not present remains a challenge. One study found that 80% of angiographically negative GI bleeding was controllable without rebleeding using conservative treatment^4^. However, rebleeding occurred in 16% of all patients, and 50% of these patients (or 8% of all patients) died of massive hemorrhage within 9 h of rebleeding onset. Therefore, death can occur in these clinical situations, which may form the basis for the need for active treatment, such as prophylactic TAE, as opposed to relying solely on conservative treatment for the management of angiographically negative GI bleeding.

To our knowledge, no published studies have focused on the clinical outcomes of QS-GSP use for prophylactic TAE in the management of acute arterial GI bleeding. We found that using QS-GSPs for prophylactic TAE can be safe and effective for bleeding control in the context of angiographically negative GI bleeding. Clinical success was achieved in 90% of patients, which was higher than the 80% of patients for whom bleeding control was achieved through conservative treatment in a previous study^4^. The high success rate is thought to be the effect of prophylactic TAE with QS-GSPs. The time it takes for arterial thrombosis to occur after GSP embolization may vary depending on various factors, such as the location of embolization or the presence of coagulopathy, but a short dissolution time of less than 4 h seems sufficient to cause thrombosis in the target artery. Hemostasis is achieved through clot formation within the arteries, which then extends to the bleeding focus. Given that the vasa recta of the small bowel are less than 1 mm in diameter^13^, thrombosis ensues quickly in these vessels.

In this study, there were no instances of TAE-related bowel ischemia or infarction. It is known that an acute, complete disruption of enteric blood supply leads to irreversible mucosal ischemia within 6 h, accompanied by cell energy loss, leukocyte infiltration, and the formation of oxygen radicals^14^. Ischemic complications of the lower GI tract can also arise from the use of ordinary GSPs. Bua-Ngam et al.^10^ used ordinary GSPs for 27 patients with lower GI bleeding, and 4 patients (14.8%) developed ischemic complications. Of the other 20 patients who underwent ordinary GSP embolization for lower GI bleeding, 1 (5%) died from small bowel infarction^11^. Moreover, even when embolization is performed to the level of the vasa recta using QS-GSPs, it is thought not to cause bowel ischemia because QS-GSP are absorbed within 4 h. Therefore, QS-GSPs are considered safe for use even in the presence of active bleeding when selective embolization is not possible and the embolization of multiple vasa recta is unavoidable. A study in which QS-GSPs were used for genicular artery embolization found that skin color changes occurred in 51% of 91 procedures, resolving within 3–5 days; this is thought to have been due to arterial recanalization after QS-GSP biodegradation^12^.

The appropriate size of embolic agent particles is crucial: if they are too small, they may cause ischemic bowel complications; if they are too large, they could diminish the embolization effect. The QS-GSPs used in this study, measuring 150–350 or 350–560 µm, are deemed ideal for occluding the vasa recta of the small bowel, which typically measure 560–770 µm in diameter^13^. Theses QS-GSPs are larger than the 40–60-µm Gelfoam powder well known to induce ischemic complications; therefore, the risk of intestinal ischemia or infarction is thought to be low.

Our study had several limitations. First, this study employed a retrospective design and included a small sample size. Further studies are needed with more patients to clarify the safety and efficacy of QS-GSPs for treating GI bleeding. Second, it was difficult to determine which particle diameter was the most appropriate because QS-GSPs of various diameters were not used.

In conclusion, prophylactic TAE using QS-GSPs has demonstrated safety and efficacy in the management of angiographically negative GI bleeding. Future research should include a prospective, randomized comparison between QS-GSPs and conventional GSPs to further validate these findings.

## Materials and methods

Due to the retrospective nature of the study, the need of informed consent was waived by Asan Medical Center institutional review board (IRB number 2023-2007). All research was performed in accordance with the Declaration of Helsinki. The study included 10 patients (7 men; mean age, 64.3 years ± 8.3; range, 47–75 years) with angiographically negative acute nonvariceal GI bleeding who underwent prophylactic TAE with QS-GSP at a single tertiary center between February 2021 and September 2023. Acute nonvariceal GI bleeding encompassed nonvariceal sources of hematemesis, melena, or hematochezia that occurred within 24 h before angiography. Variceal bleeding was excluded based on a review of the patients’ clinical information, radiologic findings, or endoscopic findings.

Electronic medical records, laboratory findings, and endoscopic and/or radiologic features were reviewed. The following data were collected for each patient: clinical data, including symptoms and signs of GI bleeding; underlying diseases such as chronic kidney disease, diabetes mellitus, or hypertension; coagulopathy; hemodynamic instability; the cause of GI bleeding; the interval between GI bleeding and TAE onset; history of previous treatment; angiographic findings and TAE details; hemoglobin levels before and within 2 days after TAE; clinical outcomes (focusing on rebleeding); and procedure-related complications.

### TAE procedure

Angiography and/or TAE procedures were performed by 1 interventional radiologist with 25 years of clinical experience in endovascular intervention. Via the common femoral artery approach, a 5-French catheter (RH catheter; Cook, Inc., Bloomington, IN, USA) was introduced over a 0.035-inch guide wire (Radifocus; Terumo, Tokyo, Japan). When femoral access was not feasible, the radial artery was accessed with a 125-cm, 5-French catheter (Performa Transradial Angiographic Catheter, Merit Medical, South Jordan, UT, USA). Celiac and/or superior mesenteric angiography was performed primarily for upper GI bleeding. Superior and inferior mesenteric angiography was performed depending on the location of the bleeding focus. Superselective angiography was subsequently performed using 2- to 2.4-French microcatheters (Progreat; Terumo, or Radiomate; S&G Biotech, Yongin, Korea) to reveal the bleeding site according to the location depicted on computed tomography (CT) and endoscopy, if available.

In the absence of active bleeding, prophylactic embolization was performed, targeting the probable bleeding focus based on the location of bleeding or probable bleeding pathology on endoscopy or CT scans. QS-GSPs (K-IPZA®; Engain, Hwaseong, Korea) were used as the embolic agent. QS-GSPs dissolve completely within 4 h after contacting saline or blood.

### Definitions and analysis

Coagulopathy was defined as a PT-INR (prothrombin time-international normalized ratio) > 1.5 or thrombocytopenia with a platelet count < 50,000/μL. Hemodynamic instability was defined as systolic blood pressure < 90 mm Hg. Technical success was defined as occlusion or stasis of blood flow in the target artery on the angiogram obtained immediately after TAE. Clinical success was defined as the cessation of bleeding symptoms or the clearing of nasogastric aspirate with hemodynamic stability during the 72 h following the TAE, without embolization-related major complications, such as bowel ischemia or infarction. Rebleeding was defined as another episode of bleeding at the same site 72 h after the initial TAE and requiring additional treatment.

Electronic medical records, follow-up endoscopy reports, and post-TAE CT scans were reviewed to assess for the presence or absence of procedure-related complications, such as bowel ischemia or infarction. Procedure-related complications were defined according to the clinical practice guidelines of the Society of Interventional Radiology. Major complications were defined as those necessitating further treatment or prolonged hospitalization, and minor complications were defined as those that resolved spontaneously^15^. The Wilcoxon signed-rank test was used to compare hemoglobin levels before and after TAE. Statistical analysis was performed using SPSS Statistics for Windows, version 23 (IBM Corp., Armonk, NY, USA). *P* values < 0.05 were considered statistically significant.

## Data Availability

The datasets generated and/or analysed during this study are available from the corresponding author upon reasonable request.
